# The Effect of Milk Protein on the Biological and Rheological Properties of Probiotic Capsules

**DOI:** 10.4014/jmb.2008.08007

**Published:** 2020-10-08

**Authors:** Bum Ju Kil, Sung Jin Yoon, Cheol-Heui Yun,, Chul-Sung Huh

**Affiliations:** 1 Biomodulation major and Center for Food and Bioconvergence, Seoul National University, Seoul 08826, Republic of Korea; 2 Department of Food Science and Biotechnology, Dongguk University-Seoul, Goyang 1036, Republic of Korea; 3 Department of Agricultural Biotechnology and Research Institute of Agriculture and Life Sciences, Seoul National University, Seoul 08826, Republic of Korea; 4 Institute of Green-Bio Science and Technology, Seoul National University, Pyeongchang-gun 2535, Republic of Korea; 5 Graduate School of International Agricultural Technology, Seoul National University, Pyeongchang-gun 234, Republic of Korea

**Keywords:** Probiotics, temperature-sensitive, milk-probiotic capsules

## Abstract

Probiotics are often infused into functional foods or encapsulated in a supplement form to maintain a healthy balance between the gut microbiota and their host. Because there are milk-based functional foods such as yogurt and cheese on the market, it has been suggested that milk-based probiotics could be incorporated into skim milk proteins in a liquid capsule. Skim milk is mainly composed of casein and whey protein, which create a strong natural barrier and can be used to encapsulate probiotics. In this study, we compared the encapsulated probiotics prepared with milkbased concentrated cell mixtures using commercial probiotics. Probiotic capsules were emulsified with skim milk proteins using vegetable oil to form a double coating layer. The product was heatstable when tested using a rheometer. The survival rate of the milk-based probiotic cells in the lower gastric environment with bile was significantly higher than commercial probiotics. Thus, milkencapsulated probiotics exhibited greater efficacy in the host than other types of probiotics, suggesting that the former could be more viable with a longer shelf life under harsh conditions than other form of probiotics. Our findings suggested that, compared with other types of probiotics, milkbased probiotics may be a better choice for producers and consumers.

## Introduction

In the development of functional foods, an effective coating technique determines the success of the product [1]. Probiotics are live microorganisms known to impart health benefits when ingested in sufficient quantities [2]. According to recent studies, probiotics confer various health-promoting effects, such as modification of gut microbiota, improvement of epithelial barrier integrity, and anti-inflammatory effects [3]. Every host species requires different strains of several bacterial species to protect its own health. It has been suggested that 1 × 10^6^–10^7^ CFU/ml of probiotics is the daily requirement for humans [4]. In this study, *Lactobacillus salivarius* strain W13, isolated from a weaning piglet, was cultured for probiotic preparation. In a previous study, *L. salivarius* strain W13 elicited antimicrobial activity against the pathogens—*Escherichia coli* K88 and Salmonella enterica serovar Typhimurium [11]. Reuterin-producing *Lactobacillus reuteri* LRT18 isolated from a pig was part of the trial, because it was previously shown to possess antimicrobial properties as when used as a probiotic [5, 6].

Dairy based foods require encapsulating to maintain freshness. Gum products are often used for this purpose [7]. However, skim milk can also be used to encapsulate the material and its cell concentrate. The milk-concentrate-cell-mixture possesses a low viscosity similar to calcium alginate, which is also effective for microencapsulation. Thus, milk concentrate-cell-mixture exhibits a higher survival rate in the lower gastric environment than is observed in free cells [8]. The gelling material is over 100 μm in size because the density of encapsulation is low [9]. Capsule sizes are quite variable, and the size range of encapsulated probiotics differs between the matrix and reservoir types. The matrix-type utilizes spray drying, emulsification, and extrusion, whereas the reservoir type uses co-extrusion, and spray coating [10]. In the previous probiotic encapsulation experiments, we developed phthalyl inulin tablets [11]. Tablet-style, coating, and the milk-concentrate-cell-mixture improved the survival rate of probiotics under acidic gastric conditions; however, if the functional food additive requires greater probiotic survival properties in the future, different forms like liquid coating might be available. Moreover, whether the preparation is in a tablet or liquid form, the appropriate coating is paramount for achieving the best results.

Dairy products typically require casein microcapsules for laminating probiotic microorganisms. However, in this study, milk proteins created a liquid encapsulant for probiotics because appropriate biopolymers already exist in food [12]. Probiotics are heat sensitive because the core material, milk proteins, can be denatured when exposed to heat [13]. Therefore, the addition of rennet was important to create a more heat-stable coating mixture. In conclusion, liquid-encapsulated milk proteins have better survival activity under high-temperature conditions.

## Materials and Methods

### Culture Conditions before Encapsulation

*L. salivarius* W13 and *L. ruteri* LRT18 strains from Kangwon University, Chunchun, Korea were obtained in a frozen form for use as probiotic microbial cells [5, 6]. *L. salivarius* strain W13 (deposited name: strain KLW001) was used to establish conditions for cultivation and lyophilization. Before encapsulation, the following were prepared in the fermenter: 85 g/l yeast extract, 20 g/l maltose, 6 g/l sodium acetate anhydrous, 1.4 g/l monopotassium phosphate, 1.0 g/l dipotassium phosphate, 1.0 g/l sodium citrate monobasic, 0.2 g/l magnesium sulfate heptahydrate, 0.02 g/l manganese, 0.01 g/l ferrous sulfate heptahydrate, 1.2 ml/l polysorbate 80, and 0.5 ml/l antifoam 204. The pH was set to 7.0 ± 0.1 with 3.0 mol/l NaOH solution. All materials were obtained from Sigma-Aldrich (USA) or Difco (USA). Viable cell numbers (CFU/ml) were determined after incubation for 18 h (stationary phase).

### Encapsulation

Skim milk powder was obtained from Difco. Rennet added to the preparation was obtained from Chr. Hansen (Denmark). A fresh stock solution of rennet was prepared daily by diluting 1 g of rennet preparation in 4 g double-distilled water [14]. Sunflower oil was obtained from a local store. The probiotic milk-concentrate-cell-mixture was incubated at 4°C with 400 μl of the rennet stock solution for 1 h to create casein micelles. Following incubation, 180 ml 10% (w/v) CaCl_2_ solution was added to the mixture, and the encapsulation process was subsequently initiated. After CaCl_2_ addition, the skim milk-cell-concentrates were combined with the rennet mixture, added to 150 g of tempered vegetable oil at 4°C in a 200 ml flask, and then magnetically agitated for five minutes to emulsify the oil into the mixture. The which skim milk droplets became gel particles. The temperature was increased to 40°C for 15 min to strengthen the encapsulating process. Encapsulation of probiotic cells formed a milk protein matrix [10, 14].

### Measurement of Rheological Properties

Rheological properties of the milk protein-probiotic capsule mixture were determined under steady and dynamic shear, as described in a previous study [15] using a rheometer (HR-3 Discovery Hybrid Rheometer, TA Instruments, USA).

### Acid Resistance

Acid resistance of the microencapsulated probiotic organisms was evaluated and compared to a free probiotic organism, which served as a control. Briefly, MRS broth (de Man, Rogosa, and Sharpe) was adjusted to pH 3.0 using 5.0 M HCl. Then, 50 μl (1%) of cultures were inoculated into 5 ml MRS broth and 10^10^ CFU/ml of each probiotic strain prepared in MRS broth for 18 h was inoculated into the modified MRS broth and incubated at 37°C for up to 2 h. The samples were taken at 30 min intervals for enumeration. Acid tolerance was determined by comparing the final plate count after 2 h with the initial plate count taken at zero hour. Acid tolerance tests were repeated three times to estimate the average and standard error.

### Bile Resistance

Bile resistance of the lactic acid bacteria was estimated using 50 μl (1%) of overnight culture (18 h) inoculated into 5 ml MRS broth and supplemented with 0.3% bovine bile (Ox-gall, Difco) or without bile (control) and incubated at 37°C for 12 h under aerobic conditions. Inoculation density was determined by incubation time. The treatment and control groups, each conducted in triplicate were compared by measuring the optical density for detecting cell growth between the two groups. The survival rate in the bile salt was calculated using the following formula:

Relative bile tolerance rate = (OD_600_ nm of 0.3% ox-gall MRS culture in the treatment group)/(OD_600_ nm of 0.3%ox-gall MRS culture in the control) [16].

### Heat Tolerance

Heat tolerance of the free and encapsulated probiotic bacteria was analyzed by exposing them to 65°C, or 75°C for up to 1 h. Each sample and 1 g of microcapsules was inoculated into 9 ml of phosphate-buffered saline in a 15 ml Falcon tube (BD, USA). One ml of free probiotic cells was used as a control. Heat tolerance was determined by comparing the final plate count after 30 min, and 1 h of heat treatment with the initial plate count at 0 h. All heat tolerance tests were repeated three times.

### Measurement of Water Activity

The water activity of the milk protein-probiotic capsule mixtures was determined using a water activity meter (Cole-Parmer, US/G-59800). Freeze-dried milk protein-probiotic mixture capsules were tested using a water activity meter. All water activity tests for the milk protein-probiotic mixtures were repeated three times to estimate the average and standard error.

### Statistical Analyses

Results are reported as the mean value with a standard deviation of triplicate analyses. The analysis of variance and Duncan’s tests were used to establish significant differences at a 0.05 significance level. Statistical analyses were performed using SPSS Statistics v.18.0.

## Results

### Rheological Behavior of Probiotics in the Milk-Concentrate-Cell-Mixture

**Fig. 1:**
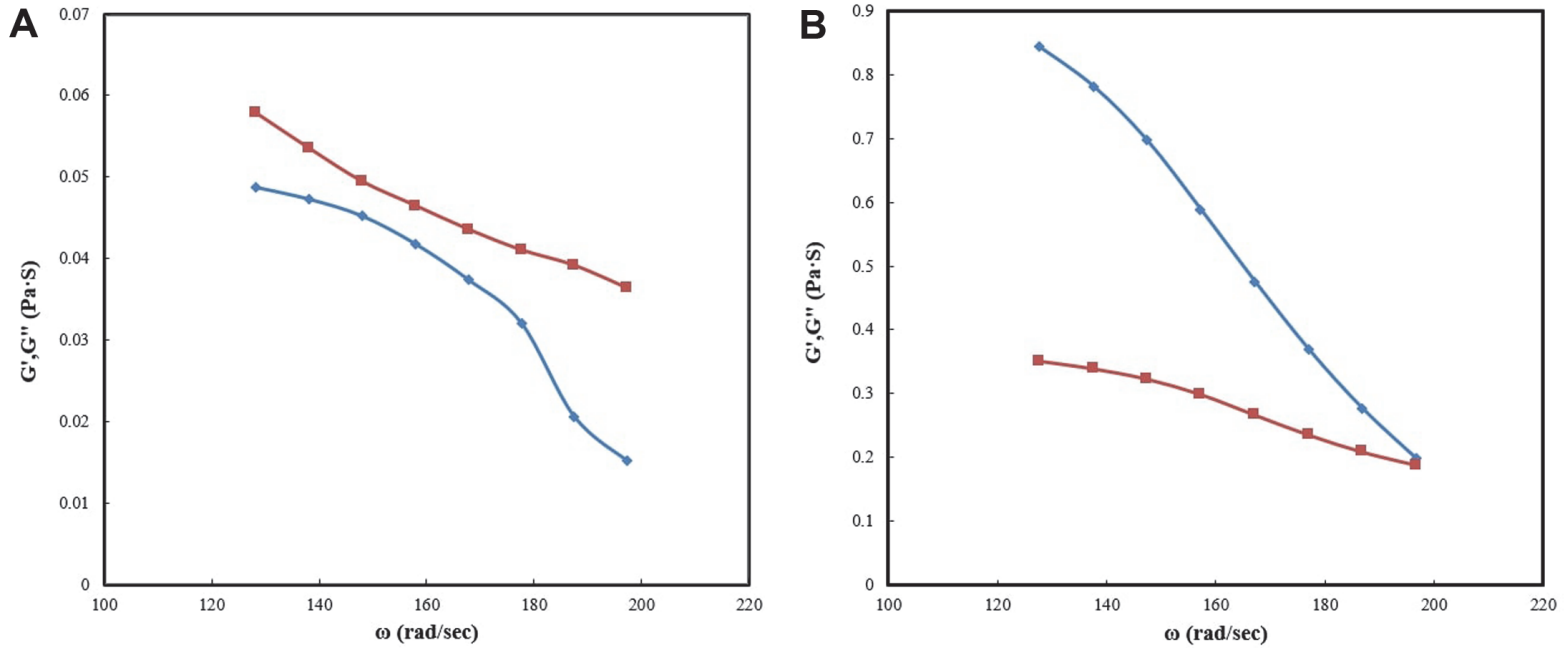
The rheological behavior lines of frequency versus storage modulus (G’) and loss modulus (G”). (**A**) Plot of log G’ (◇), log G” (□) versus ω milk protein-probiotic capsule mixtures at 25°C. (**B**) Plot of log G’ (◇), log G” (□) versus ω milk protein-probiotic capsule mixtures at 40°C.

**Fig. 2:**
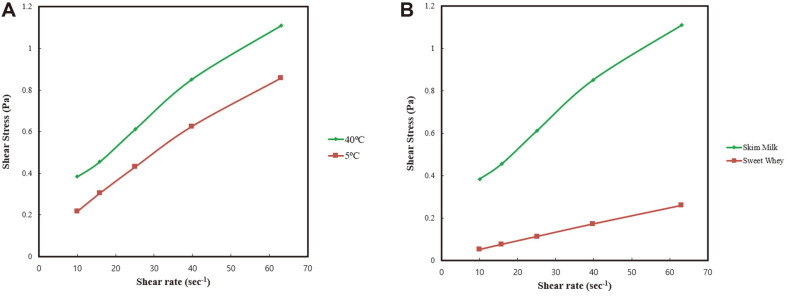
The temperature and protein source are attributed to viscosity of milk-probiotic capsules. (**A**) Shear stress-versus shear rate plots of milk-probiotic capsules at 40°C and 5°C. (**B**) Shear stress-versus shear rate plots of the milk-probiotic capsules and whey-probiotic capsules at 25°C.

Fig. 1 shows the storage modulus (G’) and loss modulus (G”). Storage modulus (G’) represents the stored deformation energy, while the loss modulus (G”) characterizes the deformation energy lost (dissipated) through internal friction when flowing. In Figs. 1A and 1B, it can be seen that G’ was higher than G” at 40°C; therefore, an emulsification temperature of 40°C is more effective than 25°C. Fig. 2 shows the shear stress-versus shear rate plots of milk-probiotic capsules at 25°C. Fig. 2A shows the differences between temperatures, while Fig. 2B shows differences between milk-probiotic and whey-probiotic capsules. The protein percentage in the skim milk was approximately 35% and 11% in sweet whey;thus, it can be inferred that protein percentage affected the shear stress-versus shear rate. We also conducted a comparison using whey protein probiotic mixtures. These results showed that milk-probiotic capsules G’ and G” were higher than the whey protein probiotic capsules G” and G” (data not shown), indicating that the milk protein source affects the rheological properties of the milk-concentrate-cell-mixture.

### Survival of Probiotic Cells in an Acidic Gastric Environment

A greater survival rate was found for the milk-probiotic mixture compared to that of free probiotics, following exposure to a simulated gastric juice environment. *L. salivarius* W13 and *L. ruteri* LRT18 both expressed gastric juice resistance. Table 1 shows the differences in survival rates between the milk-probiotic mixture and the free probiotics. These differences were directly related to the coating, as all probiotics that were coated exhibited a higher survival rate than the free probiotics. Free *L. salivarius* W13 cells experienced a 1.63 log CFU/ml reduction, while the encapsulated cells experienced a 0.76 log CFU/ml reduction. Free *L. ruteri* LRT18 cells were reduced to 1.3 log CFU/ml, while the encapsulated cells were reduced to 0.45 log CFU/ml, indicating that the free cells lost twice as many viable cells compared to the encapsulated cells. This increase in survivability would offer more significantly beneficial effects such as improving gut conditions in humans [17]. Probiotics coated with alginate achieved the highest survivability [18, 19]. Results showed that milk-probiotic mixture capsules were effective tools for improving gastric juice resistance and survivability, thereby enhancing product quality.

### Survival of Probiotic Cells under Bile Condition

The effect of the ox-gall powder on the viability of the free probiotic bacteria is presented in Table 2. All probiotics exhibited a loss of viability when exposed to 0.3% (w/v) ox-gall. However, it was revealed that the encapsulation of probiotic bacteria could protect these cells under bile conditions. *L. salivarius* W13 exhibited a higher survival rate than *L. ruteri* LRT18. Studies using a 0.3% bile salt concentration have shown that encapsulated probiotic bacteria could survive better than free probiotic cells [20]. Notably, compared with the low gastric conditions, bile conditions produced the most pronounced effects. Survivability of *L. salivarius* W13 and *L. ruteri* LRT18 was less than 10%, while that of the milk-probiotic mixture was over 30%, thus demonstrating that the survivability of the encapsulated milk probiotics showed increased resistance to bile conditions.

### Survival of Probiotic Cells at Elevated Temperatures

**Fig. 3:**
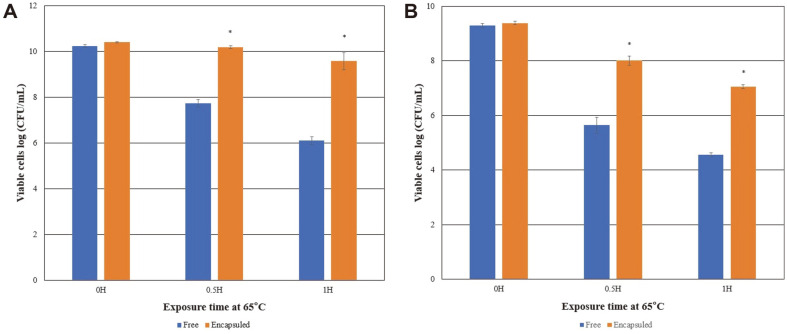
Effects of exposure to 65°C on the viability of *L. salivarius* W13 (A) and *L. ruteri* LRT18 (B). The data represent the means and standard errors of duplicates (*p < 0.05).

**Fig. 4:**
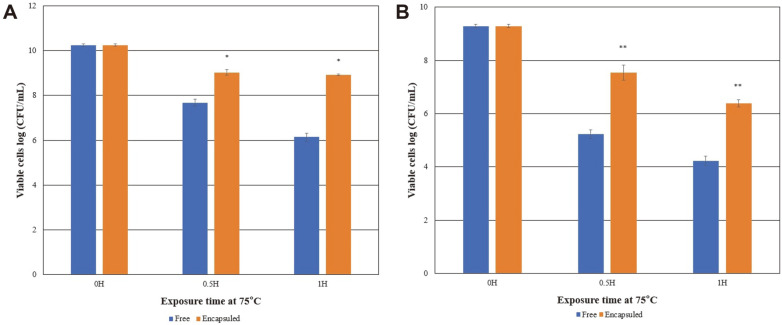
Effects of exposure to 75°C on the viability of *L. salivarius* W13 (A) and *L. ruteri* LRT18 (B). The data represent the means and standard errors of duplicates (*p < 0.05, **p < 0.01).

Milk-concentrate-cell-mixture encapsulation of probiotic cells at high temperatures resulted in more viable cells than the free probiotic cells under high-temperature conditions. The casein protein matrix protected probiotic cells at temperatures between 65°C– and 75°C for 30–60 min. *L. salivarius* W13 proved to be more heat resistant than *L. ruteri* LRT18 (Fig. 3). Both *L. salivarius* W13 and *L. ruteri* LRT18 exhibited enhanced heat tolerance following milk-concentrate-coating. Figs. 3 and 4 show that the milk-concentrate-cell-mixture imparted protection to probiotic cells at high temperatures. Thus, skim milk encapsulation improved both viability and quality of probiotics under unfavorable temperatures.

### Measurements of Water Activity after Freeze-Drying

 The freeze-dried milk protein-probiotic mixture capsules showed a water activity of 0.101 ± 0.002. This result reveals that probiotics can remain in a stable condition for an extended period.

## Discussion

This study utilized skim milk as a coating material for probiotics. In some nations, there have been issues involving the use of ordinary encapsulation materials such as alginate and gum in dairy products, therefore, milk proteins have been tested to solve these issues [10, 14]. Coating probiotics can improve heat survivability [21]. Milk protein is composed of casein and whey proteins, and the coagulation of these proteins creates a natural barrier that encapsulates and protects the liquid probiotic. Casein possesses an encapsulation efficiency of approximately 42% [22]. The protective effect due to the encapsulation of probiotic cells is attributed to the creation of a physical barrier against harsh external conditions. Optimal coatings protect the capsules from low pH conditions, remarkably cheddar cheese possesses these qualities at pH 5.5, and it could be used as a coating material for probiotics [23]. Therefore, if the information provided by this study is applied to cheese technology, cheese could be a viable candidate for probiotic encapsulation. A previous study fixed *Bifidobacterium bifidum* using an emulsifier to create an icy gel [24]. The skim milk encapsulation method significantly improved the survival rate of probiotic microbial cells, particularly under unfavorable bile conditions. This protection correlates with the rheological behavior of the probiotic milk-concentrate-cell-mixture. The rheological behavior lines of frequency versus storage modulus (G’) and loss modulus (G”) for the milk protein-probiotic mixture capsules at 25°C revealed that the magnitudes of G’ and G” decreased with an increase in ω and that G” was higher than G’ for all values of ω with a frequency dependency. Fig. 1 depicts the characterization of the gelation process. During emulsification, G’ was higher than G” at 40°C. Based on these observations, it was revealed that higher dynamic moduli of the milk protein-probiotic mixtures existed in the emulsifier at 40°C, conferring a higher level of protection than that of the milk protein-probiotic mixtures at 25°C. Furthermore, the increasing peak and ﬁnal viscosity values could be due to the effects of temperature on the milk protein, resulting in reduced mobility of the milk protein-probiotic mixture capsules.

Moreover, the pasting temperature increased following the addition of rennet. Increased pasting temperature was associated with the inhibition of granule swelling in the presence of milk proteins. At the shear rate, the skim milk’s shear stress was higher than that of the sweet whey, which utilized binding molecules. Our previous study also evaluated the characterization of the gelation process and found that during emulsification at 40°C, G’ was higher than G”. Based on these above observations, it was revealed that the higher dynamic moduli of the milk protein-probiotic mixture in the emulsifier at 40°C imparted better protection to the cells than at 25°C. When the graph for emulsification at 40°C was studied, it was noted that the storage modulus (G’) was higher than the loss modulus (G”) for the milk-probiotic mixture capsules at 25°C.

Coating is performed to protect the liquid probiotic bacteria within the milk. Probiotic mixture capsules also protect the bacteria during the drying process, which is the last stage of microencapsulation. Freeze-drying can enhance shelf-life more than liquid encapsulation. Therefore, the water activity is an important factor during storage; a water activity of 0.1 would allow the probiotic to remain in a stable condition for an extended period [25].

## Conclusion

Temperature was found to be a crucial factor when setting up encapsulation protocols for probiotics. The survivability testing clearly showed that milk-encapsulated probiotics had superior survival rates in the low pH gastric environment and in a broth that contained bile. Furthermore, encapsulation enhanced the survivability of probiotic cells at high temperatures. Collectively, the information found in this study may contribute to the development of other novel encapsulation methods.

## Figures and Tables

**Table 1:** Effect of low pH (pH 3.0) on the viability of probiotic bacteria.

Strain	Form	Viable Cell count (log CFU/mL)	

		0 h	2 h
*L. salivarius* W13	Free	10.29 ± 0.03^a^	8.66 ± 0.22^c^
	Encapsulated	10.18 ± 0.15^a^	9.42 ± 0.10^b^
*L. ruteri* LRT18	Free	9.23 ± 0.03^a^	7.93 ± 0.02^c^
	Encapsulated	9.25 ± 0.01^a^	8.80 ± 0.28^b^

Mean values in the same column with different letters are significantly different (*p* < 0.05).

**Table 2:** Bile tolerance of probiotic bacteria.

Strain	Form	Survival rate (%)
*L. salivarius* W13	Free	7.16 ± 0.73^b^
	Encapsulated	37.2 ± 2.43^a^
*L. ruteri* LRT18	Free	6.8 ± 0.75^b^
	Encapsulated	35.6 ± 3.81^a^

Mean values in the same column with different letters are significantly different (*p* < 0.05).
